# Supercritical CO_2_–Decellularized amniotic ECM hydrogel promotes immunomodulatory skin regeneration

**DOI:** 10.1016/j.mtbio.2026.103293

**Published:** 2026-06-02

**Authors:** Seongryeol Ye, Yu-Jin Kim, Jin Yoo, In Cheul Choi, Kangwon Lee, Jong Woong Park, Youngmee Jung

**Affiliations:** a Center for Biomaterials, Biomedical Research Institute, Korea Institute of Science and Technology (KIST), Seoul, 02792, South Korea; b Department of Applied Bioengineering, Graduate School of Convergence Science and Technology, Seoul National University, Seoul, 08826, South Korea; c Department of Orthopedic Surgery, College of Medicine, Korea University, Seoul, 02841, South Korea

**Keywords:** Amniotic membrane, Supercritical CO_2_ decellularization, dECM hydrogel, Immunomodulation, Skin regeneration

## Abstract

Acute ultraviolet B (UVB) exposure triggers rapid skin inflammation, characterized by immune cell infiltration, extracellular matrix (ECM) degradation, and tissue dysfunction. Although current therapeutic strategies such as hydrating dressings, hyaluronic acid/collagen formulations, and exogenous growth factors provide partial benefits, they are insufficient to promote comprehensive regeneration due to their limited ability to modulate the inflammatory and regenerative microenvironment of damaged skin. In this study, we present a decellularized porcine amniotic membrane (dPAM) hydrogel engineered via supercritical carbon dioxide (scCO_2_)–based decellularization. This method effectively removes immunogenic cellular components while preserving ECM-associated bioactive molecules, including VEGF, PDGF, IL-10, and Decorin. Notably, the dPAM hydrogel preserved matrix-bound proteins such as Decorin, which have been associated with tissue regeneration and Wnt-related signaling involved in hair follicle biology. *In vitro* studies demonstrated that the dPAM hydrogel promotes angiogenesis in endothelial cells and attenuates M1-associated pro-inflammatory responses in macrophages. Moreover, it enhanced pro-regenerative and hair follicle–associated gene responses in human follicle-derived dermal papilla cells (HFDPCs), suggesting that dPAM hydrogel may influence Wnt-related regenerative signaling. In a UVB-induced skin inflammation murine model, the hydrogel exhibited dual functionality by attenuating early immune responses and promoting tissue repair. Collectively, these findings highlight the dPAM hydrogel as a bioactive ECM-based platform capable of creating a pro-regenerative microenvironment for cutaneous repair, with potential applications in photodamage-associated skin injury and broader regenerative biomaterial strategies.

## Graphical abstract

**Figure:**
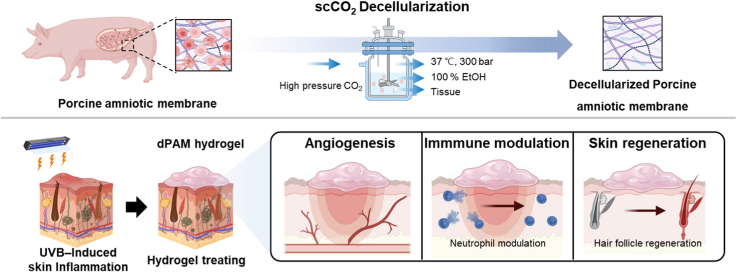

## 1 Introduction

Ultraviolet (UV) radiation is a major environmental factor that contributes to various skin pathologies, including photoaging, immunosuppression, and carcinogenesis. In particular, ultraviolet B (UVB) radiation induces pronounced inflammation and extracellular matrix (ECM) degradation due to its relatively high energy and epidermal penetration [1,2]. Beyond direct epidermal damage, UVB exposure disrupts the dermal microenvironment, including ECM organization and the hair follicle stem cell niche, both of which play critical roles in maintaining skin homeostasis and regenerative capacity [3,4]. To mitigate UVB-induced skin damage, current therapeutic approaches including hydrating dressings, natural polymer-based treatments such as hyaluronic acid and collagen, and exogenous growth factor delivery are commonly used [5,6]. These strategies mainly provide hydration, support epithelial proliferation, and stimulate angiogenesis. However, they remain insufficient for comprehensive recovery, as they are limited by short half-lives, poor bioavailability, and a lack of capacity to actively regulate immune responses. In particular, they do not address the early immune dysregulation that initiates tissue damage and impairs downstream regenerative processes [7,8].

Acute exposure to UVB radiation initiates a cascade of skin inflammation marked by immune cell infiltration and tissue damage. High-dose UVB irradiation stimulates keratinocytes and resident immune cells to release pro-inflammatory cytokines such as TNF-α, IL-1β, and IL-6. These mediators recruit neutrophils into the dermis and epidermis, amplifying inflammation and leading to further tissue injury through the production of reactive oxygen species (ROS) and proteolytic enzymes. As a consequence, the ECM undergoes extensive degradation, resulting in severe disruption of skin barrier function [1,9]. Because effective skin regeneration requires coordinated regulation of inflammation, vascularization, and ECM remodeling, recent studies have increasingly focused on ECM-based biomaterials as therapeutic platforms capable of reconstructing a regenerative microenvironment during tissue repair [10]. Among them, amniotic membrane (AM)-derived matrices are particularly attractive due to their intrinsic biological properties—including a rich composition of growth factors (e.g., VEGF, PDGF, EGF), anti-inflammatory cytokines (e.g., IL-10, HLA-G), and structural ECM components such as collagen and glycosaminoglycans [11]. These components promote epithelialization, reduce inflammation, and support tissue remodeling through diverse regeneration-related signaling pathways reported in previous studies [12,13]. In addition, amniotic ECM provides structural cues and matrix-bound bioactive molecules that can influence immune responses and dermal cell behavior, making it a promising biomaterial for inflammation-associated tissue repair [7,14].

However, despite these advantages, ECM-derived biomaterials still retain the potential to induce immune reactions by cellular components. Decellularization techniques are therefore essential to eliminate immunogenic components while preserving key structural and bioactive elements for tissue regeneration. Conventional approaches for decellularization can effectively eliminate cellular components, but often at the cost of substantial loss of bioactive molecules and disruption of ECM architecture. Detergent- or enzyme-based decellularization processes may also introduce chemical residues that compromise the biological functionality of the scaffold and reduce its regenerative potential [15]. To overcome these limitations, scCO_2_ decellularization processing has emerged as a milder alternative that preserves structural integrity and native bioactive molecules [16]. Because supercritical CO_2_ possesses gas-like diffusivity and liquid-like solvating properties, it enables efficient penetration into biological tissues while facilitating the removal of cellular components without the use of harsh detergents. This property allows improved preservation of ECM ultrastructure and matrix-associated signaling molecules that are important for tissue regeneration [17,18].

Previous studies have reported scCO_2_-based decellularization approaches for preparing tissue-derived ECM hydrogels, and amniotic membrane-derived hydrogels have also been investigated for skin repair applications. More recently, hybrid dECM-based hydrogel systems have been developed to improve the wound microenvironment and promote skin regeneration [[19], [20], [21], [22]]. However, many of these studies have focused on general wound closure, structural skin repair, composite hydrogel design, or hybrid material-based therapeutic strategies.

**Fig. 1:**
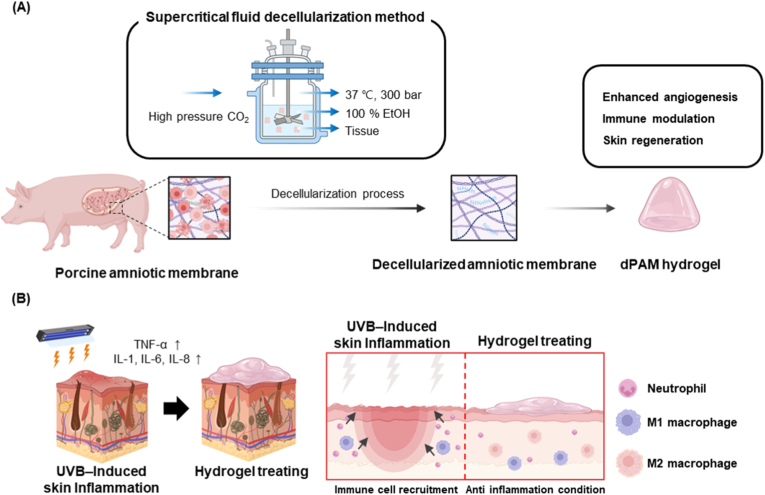
Preparation and characterization of the supercritical CO_2_–decellularized porcine amniotic membrane (dPAM) hydrogel. (A) Schematic illustration of the scCO_2_-based decellularization process used to generate the dPAM scaffold and its subsequent hydrogel formation. (B) Overview of the proposed regenerative effects of the dPAM hydrogel in modulating inflammatory responses and supporting skin repair.

In this study, we developed a single-component bioactive hydrogel derived from supercritical CO_2_–decellularized porcine amniotic membrane (dPAM), aiming to preserve native matrix-associated bioactive cues for local immunomodulation and skin regeneration (Fig. 1A). This approach resulted in substantial preservation of matrix-associated bioactive components, including key growth factors (VEGF, PDGF), anti-inflammatory cytokines (IL-10), and matrix-associated proteins such as Decorin. These preserved components are critical for coordinating tissue repair, as they provide structural support, regulate immune responses, and stimulate angiogenesis. Decorin, a small leucine-rich proteoglycan present in dermal ECM, has been reported to participate in signaling pathways associated with tissue repair, ECM remodeling, and hair follicle biology [23]. To sustain these bioactive signals within a hydrated, three-dimensional microenvironment, dPAM was processed into a hydrogel, which subsequently demonstrated multifaceted therapeutic effects. *In vitro*, it promoted angiogenesis in endothelial cells, attenuated M1-associated pro-inflammatory responses in macrophages, and enhanced immunomodulatory, pro-angiogenic, and hair follicle–associated signaling responses in HFDPCs. In a UVB-induced skin inflammation murine model, the hydrogel attenuated early immune responses and accelerated tissue regeneration, restoring both structural integrity and functional tissue repair (Fig. 1B). Collectively, these findings position the dPAM hydrogel as a bioactive ECM-based therapeutic platform capable of modulating the regenerative microenvironment during cutaneous repair, with strong potential for treating photodamaged skin and other inflammation-associated tissue injuries.

## 2 Results

### 2.1 Fabrication and structural-biochemical characterization of dPAM

**Fig. 2:**
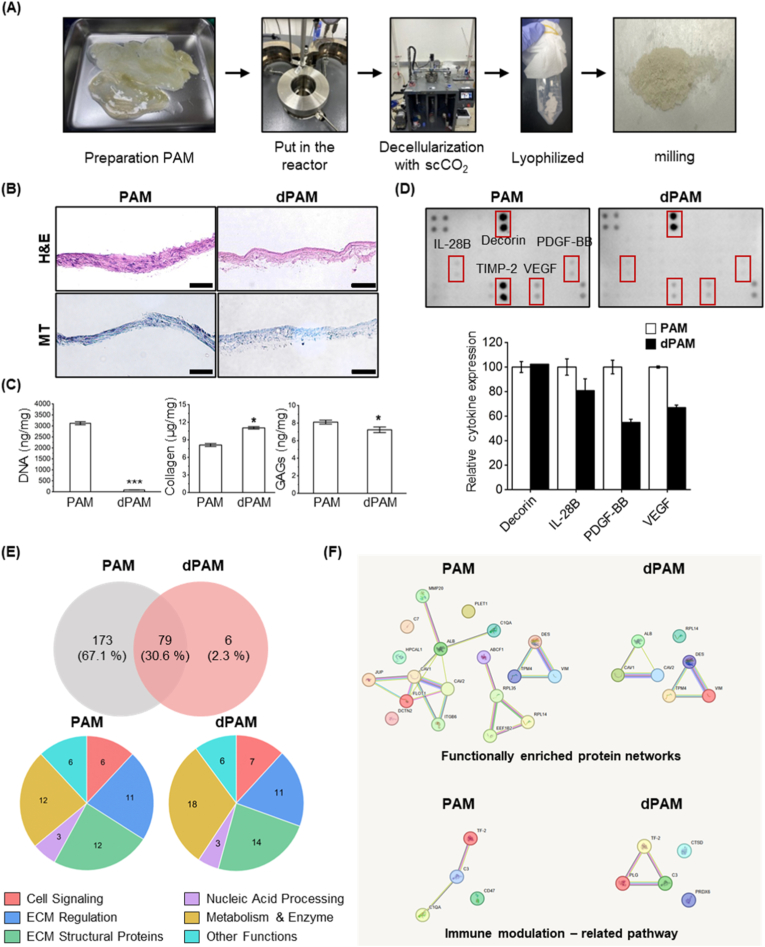
Preparation and structural/biochemical characterization of porcine amniotic membrane (PAM) and decellularized PAM (dPAM). (A) Optical images of PAM and dPAM following scCO_2_ decellularization and powder processing. (B) H&E and MT staining of PAM and dPAM before freeze-drying (scale bar = 50 μm). (C) Quantification of DNA, collagen, and GAG contents (n = 3). (D) Relative cytokine levels in PAM and dPAM (n = 2). (E) Venn diagram and functional categorization of shared and unique proteins (n = 3 biological replicates). (F) Protein–protein interaction (PPI) networks constructed from the identified proteins, highlighting clusters associated with ECM organization and immune-related processes. Statistical significance was analyzed by one-way ANOVA followed by Tukey's post hoc test. ∗p < 0.05 and ∗∗∗p < 0.001 compared to the native PAM group.

The dPAM was fabricated by isolating AM from porcine placental tissue and subjecting it to supercritical CO_2_ (scCO_2_) decellularization, a process that removes immunogenic cellular components such as residual DNA and membrane-associated proteins while preserving ECM architecture and a substantial portion of native matrix-associated bioactive molecules. Operating above 31.1 °C and 73.8 bar, scCO_2_ offers high diffusivity for deep tissue penetration and selective removal of cellular residues, while minimizing structural disruption to the ECM [16,18]. dPAM was fabricated as illustrated in Fig. 2A, and the treated tissue was subsequently freeze-dried and milled into a fine powder for long-term storage and further application.

Histological evaluation revealed that scCO_2_-based decellularization effectively removed cellular nuclei while preserving the structural integrity of the ECM (Fig. 2B). Hematoxylin and eosin (H&E) staining confirmed the absence of nuclear remnants, while Masson's trichrome (MT) staining revealed well-preserved collagen architecture, characterized by densely packed and uniformly aligned blue-stained fibers. To evaluate decellularization efficiency and ECM component preservation, we quantified the levels of DNA, collagen, and GAGs (Fig. 2C). Residual DNA content in dPAM was reduced to 83.9 ± 4.87 ng/mg, indicating efficient removal of cellular material and substantial reduction in DNA-associated immunogenic components [24]. In contrast, critical ECM components such as collagen and GAGs, key mediators of skin regeneration, were preserved. Collagen provides structural support and tensile strength for dermal repair, while GAGs maintain tissue hydration and facilitate cell migration and growth factor binding. The GAG content in dPAM (7.23 ± 0.36 μg/mg) retained approximately 90% of the amount present in native PAM (8.11 ± 0.25 μg/mg), indicating substantial preservation of ECM components associated with cellular activity and tissue repair. Interestingly, collagen content was higher in dPAM (11.14 ± 0.62 μg/mg) than in native PAM (8.27 ± 0.31 μg/mg), likely due to the relative enrichment of ECM components following removal of cellular content. This apparent increase likely reflects the relative concentration of ECM components after cellular material removal rather than new collagen formation. To further compare ECM preservation after decellularization, SDS-based decellularization was included as a conventional reference condition (Supplementary Fig. 1). Both SDS and scCO_2_ treatments markedly reduced residual DNA compared with native PAM, indicating effective removal of cellular components. However, scCO_2_-treated samples retained higher levels of GAG and collagen than SDS-treated samples. These results suggest that scCO_2_ decellularization preserved ECM-associated components more effectively than SDS treatment under the tested conditions [25,26].

Cytokine profiling was performed to assess the retention of signaling molecules associated with inflammation modulation and tissue regeneration (Fig. 2D). Cytokines such as Decorin and IL-28 B, linked to cell proliferation and anti-inflammatory activity, remained detectable in dPAM at levels comparable to those in native PAM [23,27]. Although slight reductions were observed in PDGF-BB and VEGF, both critical for cell survival and angiogenesis, their expression levels were still retained at appreciable levels following decellularization.

To further assess protein-level changes after decellularization, we performed proteomic analysis (Supplementary Fig. 2) identifying 253 total proteins, with 79 shared between PAM and dPAM. Heatmap and volcano plot analyses further visualized differentially abundant proteins between PAM and dPAM, and representative changed proteins were summarized in Supplementary Fig. 2A–C. These analyses showed reduction of several intracellular and cytoplasmic proteins after decellularization, whereas ECM-associated and matrix-interacting proteins were relatively retained in dPAM. Functional classification revealed that both PAM and dPAM retained proteins related to cell signaling, ECM regulation, and immune modulation, which are essential for comprehensive tissue regeneration (Fig. 2E). Notably, proteins such as transferrin (TF), complement component C3, and peroxiredoxin 5 (PRDX5)—associated with iron transport and cellular homeostasis support, matrix remodeling, and ROS scavenging with anti-apoptotic effects, respectively—were consistently retained in dPAM [[28], [29], [30]]. In contrast, PAM contained a broader array of cytoplasmic proteins, including those associated with intracellular metabolism, protein synthesis, and cell proliferation, many of which were eliminated through decellularization. The removal of these intracellular components may reduce potential immunogenic signals and contribute to improved biocompatibility of the resulting dPAM scaffold.

To further support the functional interpretation of the proteomic data, GO and KEGG pathway enrichment analyses were performed using the STRING database (Supplementary Fig. 2D). GO biological process analysis showed enrichment of terms associated with regulation of wound healing and antimicrobial humoral response. GO molecular function analysis showed enrichment of structural molecule activity, calcium-dependent protein binding, and collagen binding. KEGG pathway analysis further identified enrichment of complement and coagulation cascades and focal adhesion-related pathways. These findings suggest that the retained proteins in dPAM are associated with ECM-related functions, immune-related responses, and cell–matrix interactions.

Consistent with the enrichment analysis, STRING-based protein–protein interaction (PPI) analysis further highlighted differences between the two groups (Fig. 2F). PAM showed dense and heterogeneous interaction clusters reflecting diverse metabolic and proliferative functions. In contrast, dPAM exhibited clusters associated with ECM organization and immune-related processes. Importantly, immunomodulatory proteins such as C3 and TF-2 formed tightly interconnected clusters, suggesting potential coordination among complement activation, oxidative stress response, and immune cell recruitment pathways. Collectively, these findings indicate that the scCO_2_-based decellularization process not only removes immunogenic material but also retains ECM-associated protein networks that may contribute to inflammation regulation and tissue repair.

### 2.2 Surface and mechanical characterization of dPAM hydrogel

**Fig. 3:**
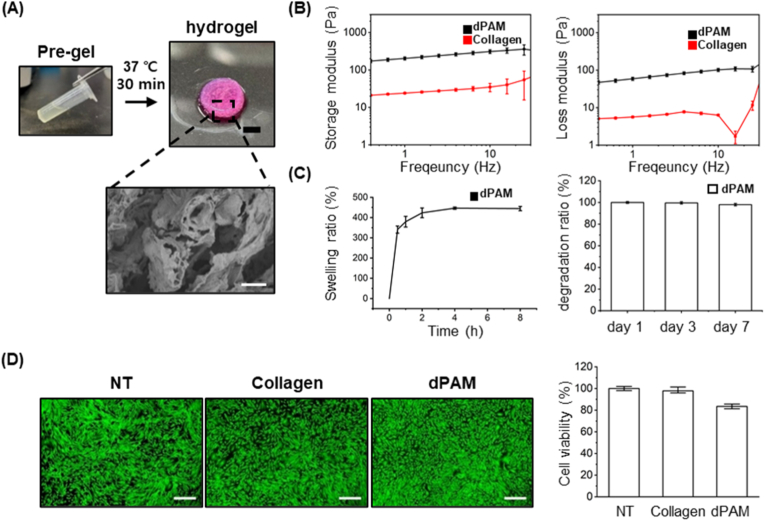
Structural, mechanical, and biocompatibility characterization of the dPAM hydrogel. (A) Photographic and SEM images of dPAM hydrogel at the optimized concentration (scale bar = 100 μm). (B) Rheological properties (storage modulus G′ and loss modulus G″) of dPAM and collagen hydrogels (n = 3). (C) Swelling ratio and degradation profile of the dPAM hydrogels (n = 3). (D) Live/dead staining and cell viability of dPAM hydrogel. Cell viability was normalized to the non-treated (NT) group (n = 4, scale bar = 500 μm). Statistical significance was analyzed by one-way ANOVA followed by Tukey's post hoc test.

A collagen-rich, thermoresponsive dPAM hydrogel was fabricated by varying the dPAM concentration, and its viscosity, macroscopic structure, and porosity were assessed through photographic imaging and SEM analysis (Supplementary Fig. 3). Based on the structural stability and interconnected porous architecture observed at different concentrations, 2% (w/v) dPAM was selected as the optimal formulation for subsequent experiments. Scanning electron microscopy (SEM) revealed a porous architecture with an average pore size ranging from approximately 40-120 μm, suitable for facilitating cell-cell interactions and diffusion of bioactive components within the hydrogel matrix (Fig. 3A) [31].

As shown in Fig. 3B, rheological measurements were conducted to evaluate the storage modulus (G′) and loss modulus (G″) of dPAM hydrogel in comparison with a collagen hydrogel (3 mg/mL), which was selected as a representative collagen-based control for dermal tissue applications based on previous reports [32]. The dPAM hydrogel exhibited a storage modulus (G′) ranging from approximately 125-300 Pa, and a loss modulus (G″) between 70 and 100 Pa. In contrast, the collagen hydrogel showed markedly lower values, with a G′ of 30-50 Pa and G″ remaining below 10 Pa. These results reflect a viscoelastic behavior of the dPAM hydrogel, with G′ consistently exceeding G″, indicating the formation of a stable elastic hydrogel network [33].

Additionally, the swelling behavior and hydrolytic degradation profile of the dPAM hydrogel were assessed in PBS (Fig. 3C). The lyophilized hydrogel rapidly absorbed water and reached a swelling ratio of approximately 350% within 30 min, followed by a plateau at approximately 400% after 4 h, indicating hydrogel saturation. Such a high water uptake capacity suggests that the hydrogel can provide a hydrated microenvironment that may facilitate nutrient diffusion and retention of bioactive molecules [34].

The degradation profile revealed that the dPAM hydrogel maintained its structural integrity without noticeable decomposition up to 7 days at 37 °C. These results indicate that the dPAM hydrogel retains its structural stability under physiological conditions during the early stage of incubation. To further assess hydrogel stability under variable wound-like microenvironmental conditions, pH-dependent degradation behavior was evaluated at pH 5, 7, and 9 for up to 7 days (Supplementary Fig. 4). The dPAM hydrogel showed comparable degradation profiles across all tested pH conditions, with no significant differences among the pH groups, indicating that its structural stability was maintained over a broad pH range. Although these results suggest pH-insensitive degradation behavior under the tested conditions, further studies examining pH-dependent swelling behavior and release kinetics of matrix-associated bioactive factors would be needed to more comprehensively predict hydrogel performance in dynamic wound environments.

### 2.3 *In vitro* evaluation of dPAM hydrogel

The dPAM hydrogel was found to contain a rich composition of structural ECM proteins, pro-angiogenic growth factors, and immunoregulatory cytokines (Fig. 2). To verify whether these bioactive components could provide therapeutic benefits, *in vitro* assays were performed. As fibroblasts are one of the most abundant cell types in the wound site and play a central role in ECM remodeling, human dermal fibroblasts (HDFs) were co-cultured with the hydrogel for 24 h to assess its biocompatibility (Fig. 3D). The dPAM group exhibited approximately 85% cell viability relative to the non-treated (NT) group, indicating favorable cytocompatibility. While minor effects on cellular metabolism may have resulted from residual salts during hydrogel preparation, the overall viability remained well within the acceptable range defined by ISO standards [35].

**Fig. 4:**
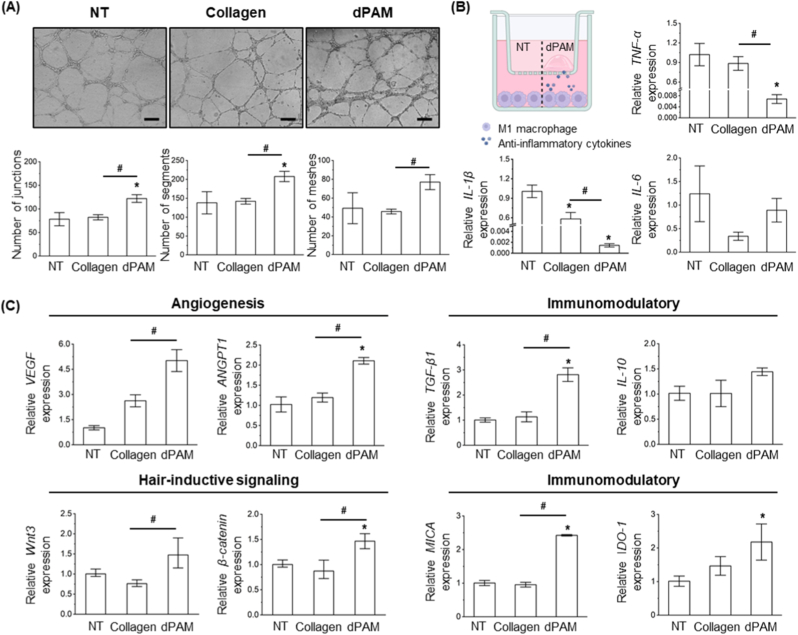
*In vitro* angiogenic, immunomodulatory, and regenerative responses associated with the dPAM hydrogel. (A) Tube formation assay using HUVECs cultured with dPAM hydrogel for 8 h. Representative images and quantitative analysis of junctions, segments, and meshes (n = 4; scale bar = 200 μm). (B) Macrophage response analysis using THP-1 monocytes treated with dPAM hydrogel for 24 h. Gene expression of pro-inflammatory (*TNF-α, IL-1β, IL-6*) was measured (n = 4). (C) Gene expression analysis in human follicle-derived dermal papilla cells (HFDPCs) co-cultured with dPAM hydrogel for 24 h. Expression levels of *VEGF, TGF-β, IL-10, Wnt3, ANGPT1, MICA, IDO-1*, and *β-catenin* were analyzed. Data are presented as relative gene expression levels normalized to the NT group. Statistical significance was analyzed by one-way ANOVA followed by Tukey's post hoc test. ∗p < 0.05 compared with the NT group, and #p < 0.05 between the indicated groups.

To further examine direct cell–hydrogel contact, live/dead staining was performed using HUVECs cultured in contact with the dPAM hydrogel (Supplementary Fig. 5). Most HUVECs remained viable under direct-contact conditions, indicating that the hydrogel provided a cytocompatible environment for endothelial cells. The subsequent functional assays were performed using an indirect transwell co-culture system as an intentional methodological approach to specifically evaluate soluble factor-mediated paracrine bioactive effects of the dPAM hydrogel, consistent with established indirect co-culture principles [36] and previous studies evaluating the immunomodulatory and angiogenic properties of hydrogel-based biomaterials using similar systems [37]. The angiogenic potential of the hydrogel was assessed using a tube formation assay with human umbilical vein endothelial cells (HUVECs) (Fig. 4A). The dPAM hydrogel-treated group demonstrated significantly enhanced tubule network formation, as evidenced by increases in the number of junctions, segments, and meshes compared with both NT and collagen hydrogel-treated groups. This effect may be associated with angiogenic factors preserved in dPAM, such as VEGF and PDGF, which are known to support endothelial cell proliferation and migration during neovascularization and tissue repair [38].

To investigate the immunomodulatory properties of dPAM hydrogel, THP-1 monocytes were polarized into M1-like macrophages by LPS and IFN-γ stimulation and subsequently co-cultured with the collagen and dPAM hydrogels (Fig. 4B). Gene expression analysis revealed a significant downregulation of *TNF-α* and *IL-1β*, key mediators of acute inflammatory signaling, in the dPAM group compared with both the collagen and control groups. *IL-6*, a cytokine that bridges inflammation and tissue remodeling, exhibited a moderate reduction rather than complete suppression. This balanced modulation may help limit excessive inflammatory signaling while maintaining IL-6–associated functions in tissue repair processes, including angiogenesis and matrix remodeling [39]. Collectively, these results indicate that dPAM hydrogel attenuates M1-associated pro-inflammatory signaling under the tested *in vitro* conditions. However, because anti-inflammatory and M2-associated markers were not comprehensively assessed in this assay, these findings should be interpreted as suppression of pro-inflammatory responses rather than definitive induction of M2 polarization.

The regenerative effects of dPAM hydrogel were further evaluated in HFDPCs, which play a central role in hair follicle maintenance and cycling (Fig. 4C). When cultured with dPAM hydrogel, HFDPCs exhibited a significant upregulation of *VEGF* and *ANGPT1*, both critical for enhancing follicular vascularization and nutrient delivery. Notably, dPAM hydrogel also increased expression of *Wnt3* and *β-catenin* in these cells. These molecules are components of signaling pathways associated with dermal papilla activity and hair follicle biology, suggesting that the dPAM hydrogel may influence regenerative signaling networks in HFDPCs [40,41]. However, because Decorin-specific loss-of-function or Wnt-inhibition experiments were not performed, the direct contribution of Decorin to Wnt/β-catenin activation could not be established in the present study.

In addition, dPAM hydrogel modulated immune-related genes in HFDPCs. *TGF-β1*, a pleiotropic cytokine involved in inflammation and matrix remodeling, was markedly elevated. Immunoregulatory markers such as IL-10 and IDO-1 were also upregulated, suggesting a shift toward a microenvironment associated with regenerative and immunoregulatory responses. IDO-1, known for its role in tryptophan metabolism, may contribute to immunoregulatory processes. In contrast, MICA, a stress-induced ligand involved in immune surveillance, may reflect cellular stress-associated immune signaling rather than direct anti-inflammatory effects [[42], [43], [44]].

Collectively, these *in vitro* results demonstrate that dPAM hydrogel supports angiogenic activity, modulates pro-inflammatory signaling, and enhances regenerative responses in dermal papilla cells. These findings highlight the potential of dPAM hydrogel to contribute to a pro-regenerative microenvironment relevant to cutaneous repair and hair follicle–associated biology.

### 2.4 *In vivo* evaluation of dPAM hydrogel

**Fig. 5:**
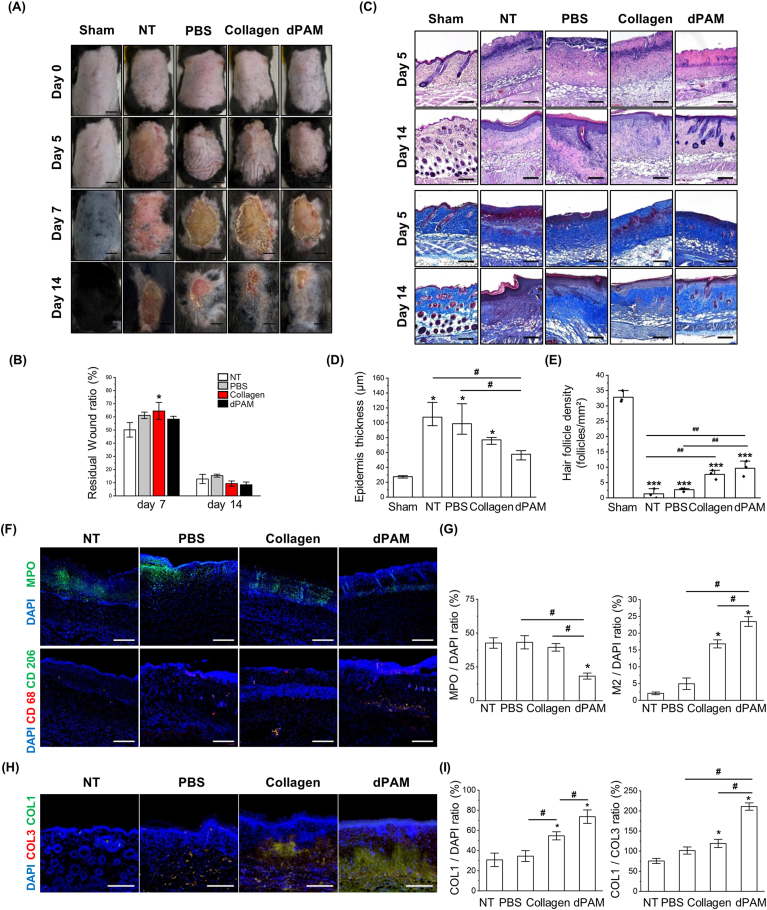
*In vivo* anti-inflammatory and wound healing effects of dPAM hydrogel. (A) Representative images of wounds treated with various methods (NT, PBS, collagen hydrogel, dPAM hydrogel) at days 0, 5, 7, and 14 post-treatments (scale bar = 5 mm). (B) Quantification of the residual wound area on day 14 for each treatment group (n = 3). (C) Representative histological images of wounds on days 5 and 14 stained with H&E and MT to evaluate epidermal and dermal tissue remodeling. (D) Measurement of epidermal thickness in H&E-stained images on day 14 for each treatment group (n = 3; scale bar = 200 μm). (E) Quantification of hair follicle density in H&E-stained images on day 14 for each treatment group (n = 3). (F) Immunofluorescence staining for neutrophils (MPO, green), macrophage marker CD68 (red), and CD206-positive macrophages (green) on day 5 (n = 3); nuclei were stained with DAPI (blue). (G) Quantification of MPO/DAPI cell ratio and CD206/DAPI cell ratio on day 5 (n = 3). (H) Immunofluorescence staining for type I collagen (COL1, green) and type III collagen (COL3, red) on day 14 (n = 3); nuclei were stained with DAPI (blue). (I) Quantification of COL1/DAPI ratio and COL1/COL3 ratio on day 14 (n = 3). Statistical significance was analyzed by one-way ANOVA followed by Tukey's post hoc test. ∗p < 0.05 compared with the NT group, and #p < 0.05 between the indicated groups.

Building upon its favorable physicochemical and *in vitro* properties, the regenerative and anti-inflammatory potential of the dPAM hydrogel was further evaluated in a UVB-induced inflammatory skin injury murine model. The single high-dose UVB irradiation condition (500 mJ/cm^2^) was selected to induce acute photodamage and sunburn-like inflammation rather than to mimic daily UV exposure. In murine UVB-induced photodamage models, 500 mJ/cm^2^ UVB has been used to induce acute skin injury characterized by epidermal pathological changes, neutrophil infiltration, and increased inflammatory cytokine expression [45]. UVB-induced skin inflammation is characterized by epidermal thickening, immune cell infiltration, and increased expression of pro-inflammatory mediators such as IL-1β, TNF-α, and IL-6, which are also involved in human sunburn responses [46]. Experimental groups included sham, NT, PBS, collagen hydrogel, and dPAM hydrogel treatments. As shown in Fig. 5A, topical application of PBS, collagen, and dPAM hydrogels maintained a moist wound environment and promoted faster visual recovery compared to the NT group. In particular, dPAM hydrogel-treated wounds exhibited a more rapid reduction of erythema and visible inflammatory signs. Quantitative wound area analysis confirmed a smaller residual wound size in hydrogel-treated groups, with the dPAM hydrogel group showing the most pronounced reduction in residual wound area by day 14 (Fig. 5B). Compared with other groups lacking ECM-derived regenerative cues, dPAM hydrogel demonstrated improved healing outcomes, highlighting the contribution of its retained bioactive matrix components.

Histological analysis further supported these findings, with H&E staining on day 5 revealing dense neutrophil infiltration in the NT, PBS, and collagen hydrogel groups, while tissues treated with dPAM and sham exhibited markedly reduced inflammatory cell infiltration (Fig. 5C). By day 14, H&E and MT staining demonstrated progressive tissue remodeling. Both collagen and dPAM hydrogel groups exhibited reestablishment of dermal-epidermal architecture, whereas the dPAM hydrogel group additionally showed the presence of hair follicle-like structures, suggesting a skin microenvironment associated with appendage-related tissue recovery.

To quantitatively assess epidermal structure, epidermal thickness was measured from H&E-stained sections (Fig. 5D). Significantly increased epidermal thickness was observed in the NT, PBS, and collagen hydrogel groups, consistent with persistent inflammatory responses. In contrast, the dPAM group showed epidermal morphology comparable to the sham control, indicating effective resolution of inflammation and tissue remodeling. Additionally, to quantitatively evaluate hair follicle-associated regenerative responses, hair follicle-like structures were manually counted from H&E-stained images on day 14 using ImageJ (Fig. 5E). The number of follicles was normalized to the analyzed area and expressed as follicles/mm^2^. The dPAM-treated group exhibited significantly higher hair follicle density than the NT and PBS groups. This quantitative result supports the histological observation that dPAM hydrogel treatment created a skin microenvironment associated with appendage-related tissue recovery, although functional hair neogenesis was not directly assessed in this study.

To further evaluate local immune responses and matrix remodeling, immunofluorescence staining was performed. Neutrophil infiltration, assessed by myeloperoxidase (MPO) staining, was significantly reduced in the dPAM group compared with all other groups (Fig. 5F). Moreover, analysis of macrophage-associated marker expression revealed an increased DAPI-normalized CD206-positive signal in the dPAM hydrogel-treated wounds, suggesting an increased presence of macrophage populations associated with pro-regenerative immune responses (Fig. 5G). These results are consistent with the previously observed enrichment of anti-inflammatory cytokines in dPAM and its *in vitro* immunomodulatory effects, supporting the hydrogel's potential role in modulating the inflammatory microenvironment. In terms of matrix remodeling, the expression of type I collagen (COL1) was substantially increased, and the COL1/COL3 ratio was elevated in the dPAM group, indicating progressive extracellular matrix maturation during the healing process (Fig. 5H). These findings were further supported by quantitative image analysis (Fig. 5I). Collectively, these findings suggest that the dPAM hydrogel supports wound closure, reduces inflammatory cell infiltration, and promotes collagen remodeling while creating a microenvironment associated with hair follicle–related regenerative responses. These effects may be attributed to the preserved ECM architecture and retained matrix-associated bioactive molecules resulting from the scCO_2_ decellularization process.

Although this study focused on local skin regeneration, several limitations should be considered. UVB irradiation may induce systemic immune responses under certain conditions; however, systemic inflammatory markers, such as serum cytokine levels and immune organ responses, were not quantitatively evaluated in the present study. In addition, only male mice were used to maintain consistency within the experimental model, and potential sex-dependent differences in UVB-induced inflammatory responses and regenerative outcomes were not assessed. Furthermore, the cytokine array and animal experiments were performed with limited sample sizes. Nevertheless, the directional consistency of findings across multiple independent assays—including histological, immunofluorescence, and gene expression analyses—supports the overall biological interpretation, although formal effect size estimation and broader generalizability would require larger cohorts in future studies. Future studies including serum cytokine analysis, spleen-related immune profiling, larger animal cohorts, targeted protein-level analyses, and both male and female animals would be valuable to more comprehensively evaluate the systemic immunological effects, biological mechanisms, and generalizability of dPAM hydrogel treatment.

## 3 Conclusion

In this study, we developed a dPAM hydrogel using a scCO_2_–based decellularization process that effectively preserves essential bioactive molecules. This hydrogel platform demonstrated regenerative and anti-inflammatory potential in both *in vitro* and in vivo models of UVB-induced skin inflammation. Through comprehensive biological assays, we showed that dPAM hydrogel enhances angiogenic activity, attenuates local inflammatory responses, supports ECM remodeling, and creates a tissue microenvironment associated with hair follicle-related regenerative responses.

These results underscore the therapeutic potential of the dPAM hydrogel not only as a wound dressing but also as a bioactive ECM-based platform capable of delivering matrix-associated signaling cues. Unlike conventional wound coverings that primarily provide a moist environment for passive healing, this hydrogel offers a biomaterial strategy that combines structural ECM support with preserved pro-regenerative and immunomodulatory factors at the injury site.

Given its biocompatibility, structural integrity, and retention of natural ECM components, the developed hydrogel platform may have potential applications in cutaneous wound management and skin repair strategies. The ability to combine structural support with biological activity provides a distinct advantage over traditional dressing materials, offering a promising approach for supporting skin regeneration and controlling inflammation in damaged tissue.

## 4 Materials and methods

### 4.1 Decellularization of porcine amniotic membrane (PAM)

Porcine placentae were purchased from Optifarm (Cheongju, Korea) and separated into the inner amniotic membrane and outer chorionic membrane. The isolated amniotic membrane was washed with distilled water (DW) and dehydrated through a graded ethanol series (30%, 50%, 70%, and 100%), each for 1 h, followed by overnight incubation in 100% ethanol. Prior to scCO_2_ treatment, the dehydrated membrane was cut into small pieces (approximately 0.5 mm × 0.5 mm). The dehydrated membrane was then placed in a reactor with 100% ethanol and subjected to scCO_2_ processing at 300–350 bar and 37 °C for 6 h, based on previously optimized conditions [18]. The processed membrane was subsequently treated with 0.3% DNase (Sigma-Aldrich, St. Louis, MO, USA) at 4 °C for 96 h, followed by washing with DW for 72 h to remove residual nucleic acids and processing reagents. Finally, the membrane was freeze-dried, milled into powder, and stored for further use. For comparison with scCO_2_-based decellularization, SDS-based decellularization was performed as a conventional detergent-based reference condition. Briefly, isolated PAM samples were washed with distilled water (DW) and incubated in 1% sodium dodecyl sulfate (SDS; Sigma-Aldrich) for 72 h under gentle agitation. After SDS treatment, the samples were washed with DW for 48 h to remove residual detergent, freeze-dried, and used only as a conventional biochemical reference for DNA, GAG, and collagen quantification.

### 4.2 Fabrication of dPAM hydrogel

dPAM powder was solubilized at a final concentration of 2% (w/v) in a pepsin solution (0.3% w/v pepsin in 0.5 M acetic acid) by stirring at 4 °C for 48 h. After complete solubilization, 10% (v/v) of 10× minimum essential medium (MEM) and 5 N NaOH were added to neutralize the solution and adjust the pH to physiological conditions. The resulting dPAM pre-gel solution was incubated at 37 °C for 30 min to induce hydrogel formation. Collagen hydrogel was prepared as a collagen-based control using rat tail collagen type I (Corning, NY, USA) at a final concentration of 3 mg/mL according to the manufacturer's protocol. After neutralization to physiological pH, the collagen solution was incubated at 37 °C to induce gelation and used for *in vitro* and in vivo comparisons.

### 4.3 Physicochemical characterization

**DNA Quantification:** Residual DNA was extracted using the DNeasy Blood & Tissue Kit (Qiagen, Hilden, Germany) following the manufacturer's protocol. The concentration of DNA was measured with a NanoDrop spectrophotometer (ND-1000, Daemyung Science, Seoul, Korea) and expressed as nanograms of DNA per milligram of tissue.

**GAG Quantification:** GAG content was determined using the dimethylmethylene blue (DMB) assay. Amniotic membrane powders were enzymatically digested in a papain solution containing 125 μg/mL papain (Sigma-Aldrich), 100 mM phosphate buffer, 10 mM cysteine (Sigma-Aldrich), and 10 mM EDTA (Sigma-Aldrich) for 18 h at 60 °C. The resulting supernatant was mixed with DMB dye solution, and absorbance was measured spectrophotometrically at 525 nm. GAG content was normalized to the dry tissue weight.

**Collagen Quantification:** Soluble collagen content was evaluated using the Sircol Collagen Assay Kit (Biocolor Life Science, Carrickfergus, UK). Briefly, membrane samples were digested in an acid-pepsin extraction reagent at 4 °C and subsequently quantified colorimetrically at 555 nm according to the manufacturer's instructions.

**Cytokine Quantification:** Cytokine profiling was performed using a porcine cytokine antibody array kit (RayBiotech, Peachtree Corners, GA, USA). Native and decellularized amniotic membrane samples were lysed in RIPA buffer by gentle rotation at 4 °C for 2 h. The lysates were then centrifuged at 13,000 × g for 20 min at 4 °C, and the supernatants were collected for analysis. The supernatants were incubated on the array membrane according to the manufacturer's protocol, and fluorescence signals were detected using a microarray scanner. Band intensities were quantified using ImageJ software.

### 4.4 Proteomic analysis

Proteomic analysis was performed to compare protein composition between native and decellularized porcine amniotic membranes. Milled tissue powders were prepared from amniotic membranes obtained from three independent porcine placentae (5 mg per sample, n = 3 biological replicates) and subjected to protein extraction, buffer exchange (10 kDa molecular weight cut-off, MWCO), and enzymatic digestion with trypsin following standard protocols. The resulting peptides were desalted, dried, and reconstituted in 0.1% formic acid prior to liquid chromatography–tandem mass spectrometry (LC–MS/MS) analysis (5 μL injection volume). Protein identification and relative quantification were performed to evaluate changes in ECM-associated and regulatory proteins following decellularization. Functional annotation and protein categorization were conducted based on Gene Ontology (GO) analysis. Graphs were generated using Origin 2022 (OriginLab, Northampton, MA, USA), and protein–protein interaction networks were visualized using the STRING database (Search Tool for the Retrieval of Interacting Genes/Proteins).

### 4.5 Hydrogel morphology and mechanical test

For scanning electron microscopy (SEM; Inspect F50, FEI, Hillsboro, OR, USA), dPAM hydrogel samples were fixed with 4% paraformaldehyde (PFA) for 3 min and sequentially dehydrated through a graded ethanol series (30%, 50%, 70%, 80%, 90%, and 100%), each for 1 h. The dehydrated hydrogels were freeze-dried for 2 days using a laboratory freeze dryer (Ilshin BioBase, Dongducheon, Korea). The dried samples were then sputter-coated with gold using an SPI-module sputter coater (SPI Supplies, West Chester, PA, USA) and imaged using SEM at an accelerating voltage of 10 kV.

The viscoelastic properties of dPAM hydrogels were evaluated using a rotational rheometer (MCR 102, Anton Paar, Austria) equipped with parallel plate geometry. Hydrogel samples were cast into discs with a diameter of 20 mm and subjected to oscillatory shear measurements. Storage modulus (G′) and loss modulus (G″) were measured over a frequency sweep under constant temperature of 25 °C.

For swelling analysis, lyophilized dPAM hydrogels were weighed, immersed in PBS at 37 °C, gently blotted at predetermined time points, and weighed again. The swelling ratio was calculated as [(swollen weight − initial dry weight)/initial dry weight] × 100.

For degradation analysis, pre-weighed dPAM hydrogels were incubated in PBS at 37 °C. At each time point, samples were collected, gently blotted to remove excess surface liquid, and weighed to calculate the remaining mass relative to the initial weight. For pH-dependent degradation analysis, hydrogels were incubated in PBS adjusted to pH 5, 7, or 9 at 37 °C for up to 7 days, and degradation was assessed at days 1, 3, and 7.

### 4.6 *In vitro* assays

#### 4.6.1 Cell culture

HDFs were cultured in Dulbecco's Modified Eagle Medium (DMEM; Gibco, Grand Island, NY, USA) supplemented with 10% fetal bovine serum (FBS; Gibco) and 1% penicillin–streptomycin (PS; Gibco). THP-1 monocytes were maintained in Roswell Park Memorial Institute 1640 medium (RPMI-1640; Gibco) containing 10% FBS and 1% PS. Human umbilical vein endothelial cells (HUVECs) were cultured in endothelial cell basal medium (EBM; Lonza, Walkersville, MD, USA) supplemented with the EGM™ BulletKit™ (Lonza), 10% FBS, and 1% PS. Human follicle dermal papilla cells (HFDPCs) were maintained in follicle cell growth medium (CellnTech, Seoul, Korea) according to the manufacturer's instructions. All cells were incubated at 37 °C in a humidified atmosphere with 5% CO_2_.

**Cytocompatibility:** HDFs were seeded at a density of 3 × 10^4^ cells per well in 24-well plates and incubated with dPAM hydrogel placed in the upper chamber of a transwell insert for 24 h. Cell viability was assessed using a Cell Counting Kit-8 (CCK-8, Dojindo, Kumamoto, Japan) according to the manufacturer's instructions. Absorbance was measured at 450 nm using a microplate reader, and cell viability was expressed relative to untreated controls.

**Direct cell–hydrogel contact live/dead staining:** To evaluate direct cell–hydrogel contact cytocompatibility, dPAM hydrogels were placed in 48-well plates, and HUVECs were seeded onto the hydrogel surface at 2 × 10^4^ cells per well. After 24 h of culture, cells were stained with calcein-AM and ethidium homodimer-1 using a live/dead staining kit according to the manufacturer's instructions. Live and dead cells were visualized using a fluorescence microscope, and representative bright-field and fluorescence images were obtained to assess direct-contact cytocompatibility.

**Tube Formation Assay:** HUVECs were seeded at 2 × 10^4^ cells per well in 96-well plates pre-coated with Matrigel (Corning, NY, USA). The upper transwell insert containing the dPAM hydrogel was introduced, and cells were incubated for 8 h. Capillary-like structures were imaged and quantitatively analyzed using ImageJ software (National Institutes of Health, Bethesda, MD, USA) with the Angiogenesis Analyzer plugin.

**Macrophage Response Assay:** THP-1 monocytes were stimulated toward an M1-like macrophage phenotype using 100 ng/mL lipopolysaccharide (LPS) and 20 ng/mL interferon-gamma (IFN-γ) for 24 h. Subsequently, dPAM hydrogel placed in the upper transwell chamber was co-cultured with the cells for an additional 24 h. Total RNA was extracted using QIAzol reagent and purified with the RNeasy Mini Kit (Qiagen). Expression of inflammatory markers (TNF-α, IL-1β, IL-6) was quantified using quantitative reverse transcription PCR (qRT-PCR).

**Follicle Co-culture Assay:** Human follicle dermal papilla cells (HFDPCs) were seeded at 3 × 10^4^ cells per well in 24-well plates and cultured in the lower chamber of a transwell system. The dPAM hydrogel was placed in the upper insert to enable indirect paracrine signaling. After 24 h of co-culture, total RNA was extracted and analyzed via qRT-PCR. Expression levels of genes associated with angiogenesis (*VEGF, ANGPT1*), immune regulation (*IL-10, TGF-β1, IDO-1, MICA*), and dermal papilla signaling (*Wnt3, β-catenin*) were quantified.

#### 4.6.2 Quantitative reverse transcription PCR (qRT-PCR) analysis

Total RNA was extracted using QIAzol lysis reagent (Qiagen) according to the manufacturer's protocol. After phase separation with chloroform, the aqueous RNA phase was collected and purified using the RNeasy Mini Kit (Qiagen). RNA concentration and purity were measured using a spectrophotometer. For reverse transcription, 500 ng to 1 μg of total RNA was converted to complementary DNA (cDNA) using SuperScript III reverse transcriptase (Invitrogen, Carlsbad, CA, USA) and random hexamer primers. Quantitative PCR was performed using Fast SYBR Green Master Mix (Applied Biosystems, Foster City, CA, USA) on a StepOne real-time PCR system or equivalent (Applied Biosystems). Primer sequences were designed using the UCSC Genome Browser and NCBI database and are listed in Table 1. All reactions were performed in triplicate. Relative gene expression levels were calculated using the 2^–ΔΔCt method and normalized to GAPDH as the internal reference gene.

**Table 1:** Primer sequences for quantitative real time PCR.

Gene	Primer	Sequence (5′-3′)
***Human GAPDH***	Forward	GTCTCCTCTGACTTCAACAGCG
	Reverse	ACCACCCTGTTGCTGTAGCCAA
***Human TNF-α***	Forward	TCTTCTCGAACCCCGAGTGA
	Reverse	CCTCTGATGGCACCACCAG
***Human IL-1β***	Forward	AATTTGAGTCTGCCCAGTTCCC
	Reverse	AGTCAGTTATATCCTGGCCGCC
***Human IL-6***	Forward	GCACTGGCAGAAAACAACCT
	Reverse	TCAAACTCCAAAAGACCAGTGA
***Human VEGF***	Forward	GAGGGCAGAATCATCACGAAGT
	Reverse	CACCAGGGTCTCGATTGGAT
***Human ANGPT1***	Forward	CAACAGTGTCCTTCAGAAGCAGC
	Reverse	CCAGCTTGATATACATCTGCACAG
***Human Wnt3a***	Forward	ATGAACCGCCACAACAACGAGG
	Reverse	GTCCTTGAGGAAGTCACCGATG
***Human β-catenin***	Forward	CACAAGCAGAGTGCTGAAGGTG
	Reverse	GATTCCTGAGAGTCCAAAGACAG
***Human TGF-β1***	Forward	TACCTGAACCCGTGTTGCTCTC
	Reverse	GTTGCTGAGGTATCGCCAGGAA
***Human IL-10***	Forward	TCTCCGAGATGCCTTCAGCAGA
	Reverse	TCAGACAAGGCTTGGCAACCCA
***Human IDO-1***	Forward	GCCTGATCTCATAGAGTCTGGC
	Reverse	TGCATCCCAGAACTAGACGTGC
***Human MICA***	Forward	CCACCAGGATTTGCCAAGGAGA
	Reverse	CTGCCAATGACTCTGAAGCACC

#### 4.6.3 *In vivo* UVB-induced skin inflammation model

All animal procedures were approved by the KIST Institutional Animal Care and Use Committee (KIST-IACUC-2022-014-5). Six-week-old male C57BL/6 mice (Orient Bio, Seongnam, Korea) were housed under specific pathogen-free (SPF) conditions with a 12-h light/dark cycle and ad libitum access to food and water. To synchronize the hair cycle, dorsal hair was removed using electric clippers followed by a depilatory cream, and UVB exposure was performed the following day. Mice were irradiated with UVB radiation at a dose of 500 mJ/cm^2^ using a calibrated UVB lamp. On the day after irradiation, treatments were initiated by topical application of PBS, collagen hydrogel, or dPAM hydrogel. For the hydrogel-treated groups, 300 μL of collagen hydrogel or dPAM hydrogel was applied once to each irradiated skin area and immediately covered with a Tegaderm film (3M, St. Paul, MN, USA) to maintain a moist environment and ensure stable contact with the treated site. The Tegaderm dressing was maintained until day 5 without hydrogel reapplication. After removal of the dressing, wound healing was monitored until day 14 without additional hydrogel treatment. Mice were photographed on days 0, 5, 7, and 14. At the end of the experimental period, animals were euthanized and skin tissues were harvested for histological and immunofluorescence analyses.

#### 4.6.4 H&E and MT staining for histological analysis

For histological evaluation, skin tissues harvested on days 5 and 14 were fixed in 10% neutral buffered formalin for 48 h at room temperature. Fixed tissues were embedded in optimal cutting temperature (OCT) compound (Leica, Wetzlar, Germany), rapidly frozen using dry ice and liquid nitrogen, and cryosectioned at 10–12 μm thickness using a cryostat (CM3050 S; Leica). Sections were mounted on glass slides and stained with H&E or MT following standard protocols. Stained sections were examined using a light microscope (Olympus, Tokyo, Japan). Epidermal thickness was quantified from H&E-stained images using ImageJ software.

#### 4.6.5 Immunofluorescence staining

For immunofluorescence analysis, cryosectioned slides were blocked with 4% bovine serum albumin (BSA; Sigma-Aldrich) for 1 h at room temperature. Sections were incubated overnight at 4 °C with the following primary antibodies: Alexa Fluor 594-conjugated anti-CD68 (1:100; Santa Cruz Biotechnology, Dallas, TX, USA), Alexa Fluor 488-conjugated anti-CD206 (1:100; Santa Cruz Biotechnology), *anti*-MPO (1:100; Abcam, Cambridge, UK), anti-collagen type I (COL1; 1:100; Abcam), and anti-collagen type III (COL3; 1:100; Abcam). After washing, appropriate fluorophore-conjugated secondary antibodies were applied, and nuclei were counterstained with DAPI (Invitrogen). Stained slides were visualized using a fluorescence microscope. Quantification of immunofluorescence images was performed using ImageJ software. MPO and CD206 signals were quantified as DAPI-normalized ratios, and collagen remodeling was evaluated using the COL1/DAPI and COL1/COL3 ratios.

### 4.7 Statistical analysis

All data are presented as mean ± standard deviation (SD). Statistical significance was determined by one-way analysis of variance (ANOVA) followed by Tukey's post hoc test. Differences were considered significant at p < 0.05, p < 0.01, and p < 0.001. All statistical analyses were performed using Origin 2020 software.

## CRediT authorship contribution statement

**Seongryeol Ye:** Conceptualization, Investigation, Methodology, Writing – original draft. **Yu-Jin Kim:** Data curation, Investigation. **Jin Yoo:** Investigation. **In Cheul Choi:** Investigation. **Kangwon Lee:** Supervision. **Jong Woong Park:** Supervision. **Youngmee Jung:** Conceptualization, Funding acquisition, Supervision, Writing – review & editing.

## Declaration of Competing Interest

The authors declare that they have no known competing financial interests or personal relationships that could have appeared to influence the work reported in this paper.

## Data Availability

Data will be made available on request.
