# Supplementary material for: Supercritical CO2–Decellularized amniotic ECM hydrogel promotes immunomodulatory skin regeneration
Source: Mater Today Bio. 2026 Jun 2;38:103293. doi: 10.1016/j.mtbio.2026.103293 (PMC13266192; doi:10.1016/j.mtbio.2026.103293)
Supplement: Multimedia component 1 [file mmc1.pdf]

## Supplementary Fig.1

(A)

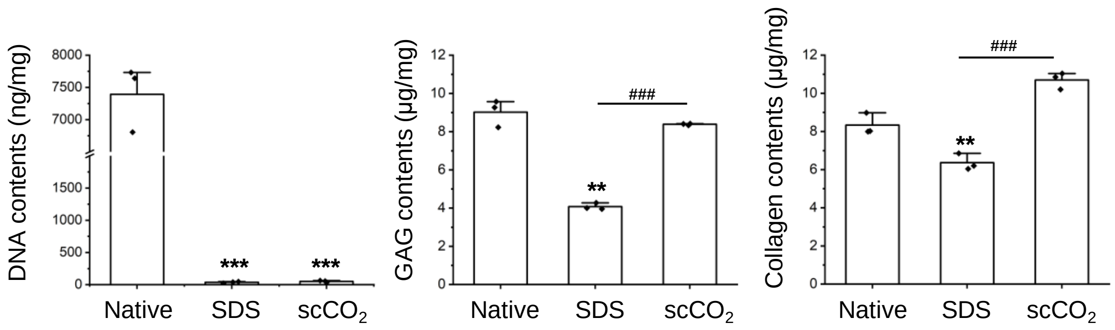


**Supplementary Fig. 1. Comparison of residual DNA and ECM component retention after SDS- and $scCO_2$-based decellularization.** DNA, GAG, and collagen contents were quantified in native PAM, SDS-decellularized PAM, and $scCO_2$-decellularized PAM (n = 3). Statistical significance was analyzed by one-way ANOVA followed by Tukey's post hoc test. **p < 0.01 and ***p < 0.001 compared with the native PAM group; ###p < 0.001 between the indicated groups.

## Supplementary Fig.2

(A) (B)

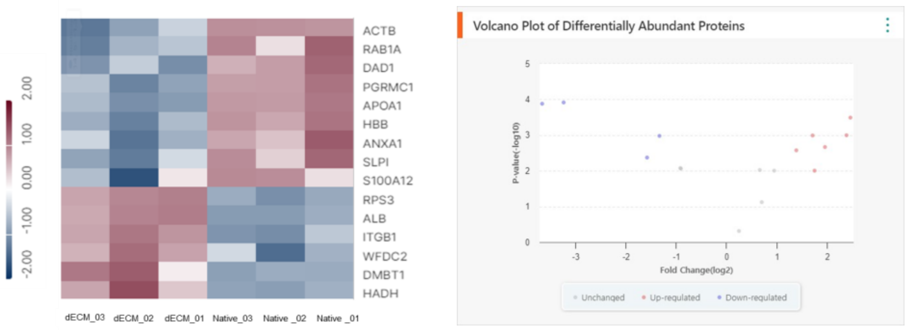


(C)

| ID | gene | fold_change | p_value | protein_id | protein_description | protein_probability | total_peptides | total_spectral_count | unique_spectral_count |
|---|---|---|---|---|---|---|---|---|---|
| 1 | RPS3 | -3.6607 | 0.00013 | Q0Z8U2 | Small ribosomal subunit protein uS3 | 1 | 11 | 39 | 39 |
| 2 | ALB | -3.23533 | 0.00012 | P08835 | Albumin | 1 | 43 | 658 | 614 |
| 3 | HADH | -1.57701 | 0.00426 | P00348 | Hydroxyacyl-coenzyme A dehydrogenase, mitochondrial | 1 | 3 | 9 | 9 |
| 4 | ITGB1 | -1.32772 | 0.00107 | Q9GLP0 | Integrin beta-1 | 1 | 11 | 47 | 43 |
| 5 | WFDC2 | -0.92478 | 0.00834 | Q8MI69 | WAP four-disulfide core domain protein 2 | 0.9992 | 2 | 8 | 8 |
| 6 | DMBT1 | -0.90349 | 0.00869 | Q4A3R3 | Deleted in malignant brain tumors 1 protein | 1 | 17 | 289 | 289 |
| 7 | CTSB | 0.25465 | 0.48524 | A1E295 | Cathepsin B | 1 | 8 | 48 | 48 |
| 8 | SLPI | 0.65969 | 0.00964 | P22298 | Antileukoproteinase (Fragment) | 1 | 10 | 75 | 75 |
| 9 | S100A12 | 0.69504 | 0.07462 | P80310 | Protein S100-A12 | 1 | 3 | 43 | 43 |
| 10 | ANXA1 | 0.95487 | 0.01005 | P19619 | Annexin A1 | 1 | 16 | 188 | 188 |
| 11 | DAD1 | 1.39067 | 0.00263 | Q29036 | Dolichyl-diphosphooligosaccharide--protein glycosyltransferase subunit DAD1 | 1 | 3 | 17 | 17 |
| 12 | PGRMC1 | 1.70731 | 0.00103 | Q95250 | Membrane-associated progesterone receptor component 1 | 1 | 9 | 36 | 36 |
| 13 | RAB1A | 1.75282 | 0.00977 | Q52NJ2 | Ras-related protein Rab-1A | 1 | 8 | 21 | 4 |
| 14 | ACTB | 1.96169 | 0.0022 | Q6QAQ1 | Actin, cytoplasmic 1 | 1 | 20 | 216 | 99 |
| 15 | HBB | 2.38948 | 0.00102 | P02067 | Hemoglobin subunit beta | 1 | 12 | 404 | 308 |
| 16 | APOA1 | 2.46069 | 0.00033 | P18648 | Apolipoprotein A-I | 1 | 15 | 180 | 180 |

(D)

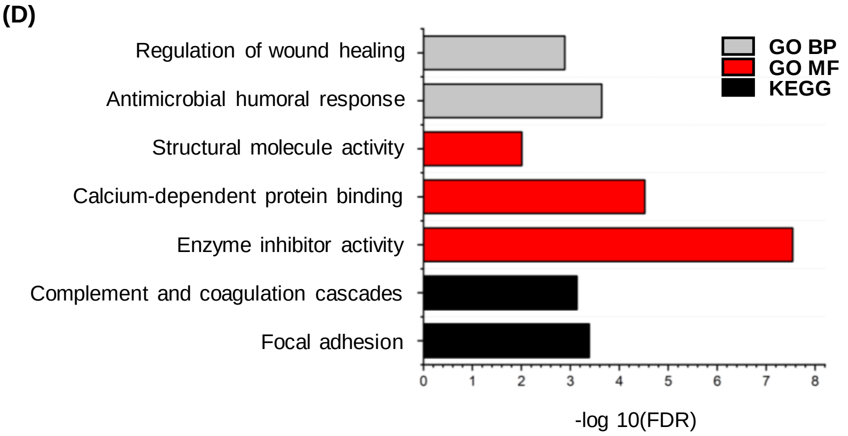


**Supplementary Fig. 2. Proteomic characterization and functional enrichment analysis of dPAM hydrogel following $scCO_2$ decellularization.** (A) Heatmap showing differentially abundant proteins between Native and dPAM groups. (B) Volcano plot representing differentially abundant proteins identified by proteomic analysis. Red and blue dots indicate upregulated and downregulated proteins, respectively. (C) List of representative differentially abundant proteins identified between PAM and dPAM samples. (D) GO and KEGG pathway enrichment analyses of proteins retained in dPAM hydrogel using the STRING database. GO biological process analysis demonstrated enrichment in pathways associated with wound healing and immune-related responses, while GO molecular function and KEGG pathway analyses revealed enrichment of extracellular matrix-associated functions and cell–matrix interaction–related pathways. Enrichment significance is presented as −log10(FDR).

## Supplementary Fig.3

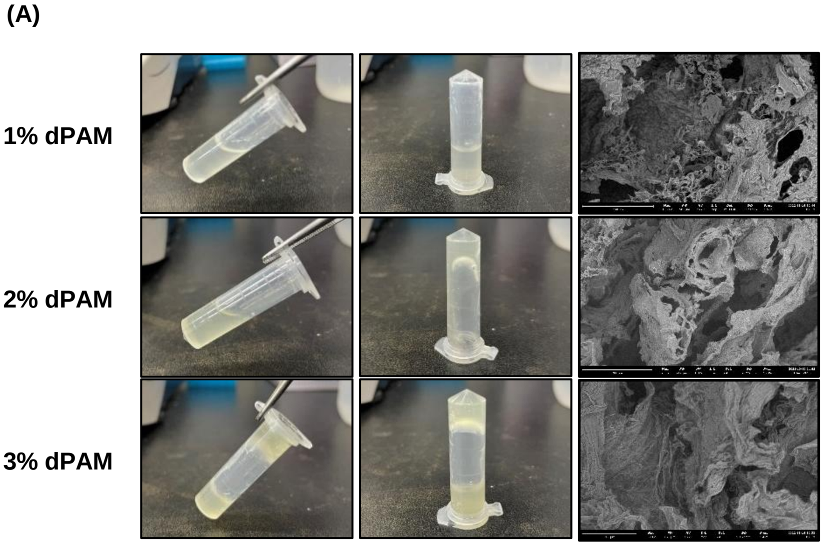


**Supplementary Fig. 3. Optimization of dPAM hydrogel concentration.** Representative images of dPAM hydrogels prepared at different concentrations (1%, 2%, and 3% w/v).

## Supplementary Fig.4

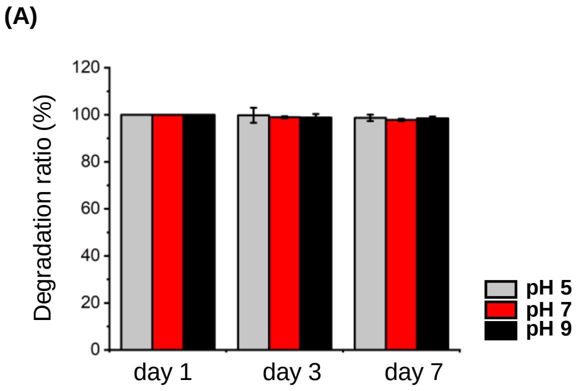


**Supplementary Fig. 4. pH-dependent degradation behavior of dPAM hydrogel.** Degradation profiles of dPAM hydrogel were evaluated under different pH conditions (pH 5, 7, and 9) at days 1, 3, and 7 (n = 3). Statistical significance was analyzed by one-way ANOVA followed by Tukey's post hoc test; ns, not significant.

## Supplementary Fig.5

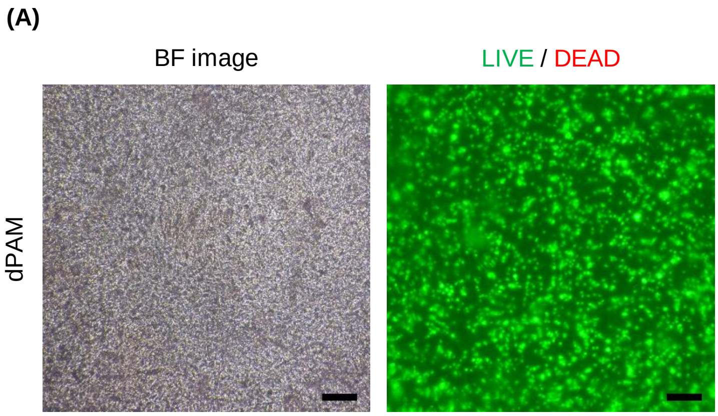


**Supplementary Fig. 5. Direct cell–hydrogel contact viability assay using HUVECs.** Representative bright-field and live/dead fluorescence images of HUVECs cultured in direct contact with the dPAM hydrogel. Live cells are shown in green and dead cells in red. Scale bar = 200 µm.
